# Improved Somatotopic Consistency of EEG Source Localization Using a Personalized Segmentation-Free Head Model

**DOI:** 10.1007/s10548-026-01217-3

**Published:** 2026-05-26

**Authors:** Yuki Tada, Akimasa Hirata, Yoshiki Kubota, Masaki Fukunaga, Toshiaki Wasaka

**Affiliations:** 1 Department of Electrical and Mechanical Engineering, Nagoya Institute of Technology, Nagoya, 466-8555 Japan; 2 Center of Biomedical Physics and Information Technology, Nagoya Institute of Technology, Nagoya, 466-8555 Japan; 3 Section of Brain Function Information, National Institute for Physiological Sciences, Okazaki, 444-8585 Aichi Japan; 4 Physiological Science Program, Graduate Institute for Advanced Studies, SOKENDAI, Hayama, 240-0193 Kanagawa Japan; 5 Core for Spin Life Sciences, Okazaki Collaborative Platform, National Institutes of Natural Sciences, Okazaki, 444-8585 Aichi Japan

**Keywords:** Electroencephalogram (EEG), Head Modeling, Source Localization, Volume Conductor Model

## Abstract

Somatosensory evoked potentials (SEPs) are widely used to assess the responses of somatosensory pathways and the central nervous system; however, the spatial resolution of EEG-based source localization is limited. This study examined whether a personalized segmentation-free head model combined with a sparse inverse solver could resolve finger-wise somatotopy without relying on functional MRI or magnetoencephalography. Sixteen healthy volunteers received electrical stimulation of the median nerve, ulnar nerve, and individual fingers, while SEPs were recorded using a 65-channel EEG. For each subject, high-resolution MRI was used to construct both conventional tissue-segmented and segmentation-free head models. Forward fields were computed using a finite difference method, and P20/N20 sources were estimated using orthogonal matching pursuit. Localization differences were evaluated as distances from reference coordinates reported in fMRI studies and compared with those obtained using a standard FEM-based pipeline with a distributed inverse solution. The segmentation-free model consistently reduced localization differences relative to literature-based reference coordinates by up to 2.9 mm. Across all stimulation targets, source-location differences (10–15 mm) were comparable to or smaller than those obtained using the conventional pipeline. Finger stimulation revealed clear somatotopic progression from the thumb to the little finger. These findings indicate that personalized segmentation-free modeling improves the spatial consistency of EEG-based source localization across multiple somatosensory targets. Rather than implying intrinsic enhancement of EEG spatial resolution, the results highlight the contribution of forward-model refinement to reducing systematic localization bias and enabling noninvasive assessment of somatotopic organization in the primary somatosensory cortex.

**Supplementary Information:**

The online version contains supplementary material available at 10.1007/s10548-026-01217-3.

## Introduction

Somatosensory evoked potentials (SEPs), which are electrical responses generated in the cerebral cortex following peripheral nerve stimulation, have long been investigated as reliable indicators for the presurgical diagnosis of brain disorders and assessment of the functional integrity of neural pathways (Perot Jr 1973; Ji et al. 2013). In clinical settings, clarifying the sources and temporal characteristics of SEPs contributes to the identification of lesion sites and understanding functional impairment. Furthermore, brain-machine interfaces (BMIs), which decode neural activity to control external devices or computers, require accurate real-time estimation of the user’s intentions (Lebedev and Nicolelis 2006). Therefore, research on the sources and temporal features of SEPs is valuable for advancing our understanding of neural pathways and brain function (Hari and Forss 1999).

Electroencephalography (EEG) is one of the most widely used techniques for SEP recording. Compared with magnetoencephalography (MEG) and functional magnetic resonance imaging (fMRI), EEG offers millisecond temporal resolution but has lower spatial accuracy. MEG is mainly sensitive to superficial tangential currents, whereas fMRI cannot resolve early SEP components. Consequently, neither modality alone can capture fast, fine-grained somatosensory responses originating from deeper cortical structures. EEG source localization further suffers from the well-known inverse problem: different patterns of cortical activity can produce similar scalp potentials (Ebersole 1994; Rice et al. 2013). Therefore, accurate forward modeling is essential for stabilizing the inverse solution and improving localization fidelity (Hallez et al. 2007).

Traditional forward models often approximate the head as a sphere or as a simplified structure composed of a few homogeneous tissue types (Yvert et al. 1997; Vorwerk et al. 2012; An et al. 2022; Depuydt et al. 2024). However, highly conductive and heterogeneous tissues, such as the cerebrospinal fluid (CSF), vasculature, and localized anatomical variations, have substantial effects on current flow. Neglecting these features can lead to inaccurate representations of intracranial electric fields and degraded localization accuracy, making anatomically realistic head models necessary for reliable analysis. Recent MRI-based methods allow voxel-wise conductivity estimation without explicit segmentation (Rashed et al. 2020b), producing smoother and more physiologically plausible transitions than previous approaches. Although these segmentation-free models improve forward-field accuracy (Moridera et al. 2021), most prior EEG studies have examined only single-site stimulation, leaving their capacity to resolve more detailed functional distinctions untested.

SEPs elicited by median nerve stimulation are widely used because they yield robust and reproducible cortical responses (Bergamaschi et al. 1993; Connemann et al. 1999). In comparison, the source localization of SEPs evoked by the ulnar nerve or individual fingers has been far less explored, despite its potential to probe the effective spatial resolution of EEG-based methods. MEG and fMRI studies have demonstrated that finger representations in the primary somatosensory cortex are spatially ordered. However, whether EEG, when combined with individualized anatomically-detailed head models and modern sparse inverse solvers, can resolve finger-wise somatotopy remains largely unknown. Demonstrating such capability would represent a meaningful methodological advance because EEG is far more accessible and cost-effective than MEG or fMRI for routine or bedside neural assessments.

Recent studies have demonstrated that segmentation-free, voxel-wise conductivity estimation can improve the physical realism of EEG forward models by reducing artificial discontinuities at tissue boundaries (Rashed et al. 2020b; Moridera et al. 2021; Hirata et al. 2024). These studies primarily focused on methodological validation of the forward solution or on single-site stimulation paradigms. However, it remains unclear whether such conductivity modeling translates into improved spatial consistency across multiple somatosensory targets, particularly at the level of individual finger representations.

Thus, the primary contribution of the present study is not the introduction of a new head-modeling algorithm per se, but a systematic evaluation of segmentation-free voxel-wise conductivity modeling in the context of multi-target SEP stimulation. In particular, we examine whether this forward-model refinement, combined with sparse source estimation, improves spatial consistency and enables resolution of finger-wise somatotopy using EEG alone.

In this study, we recorded SEPs elicited by stimulation of the median nerve, ulnar nerve, and individual fingers and estimated their cortical generators using numerical head models developed by our group. The localization accuracy was quantitatively assessed and compared with a conventional finite-element-based forward model combined with a standard distributed inverse solver (Medani et al. 2023). We hypothesized that a segmentation-free, voxel-wise conductivity head model combined with a sparse inverse solver would (i) reduce localization errors relative to conventional segmented or FEM-based pipelines and (ii) enable EEG to resolve finger-wise somatotopy at a level consistent with MEG and fMRI.

## Materials and Methods

**Fig. 1:**
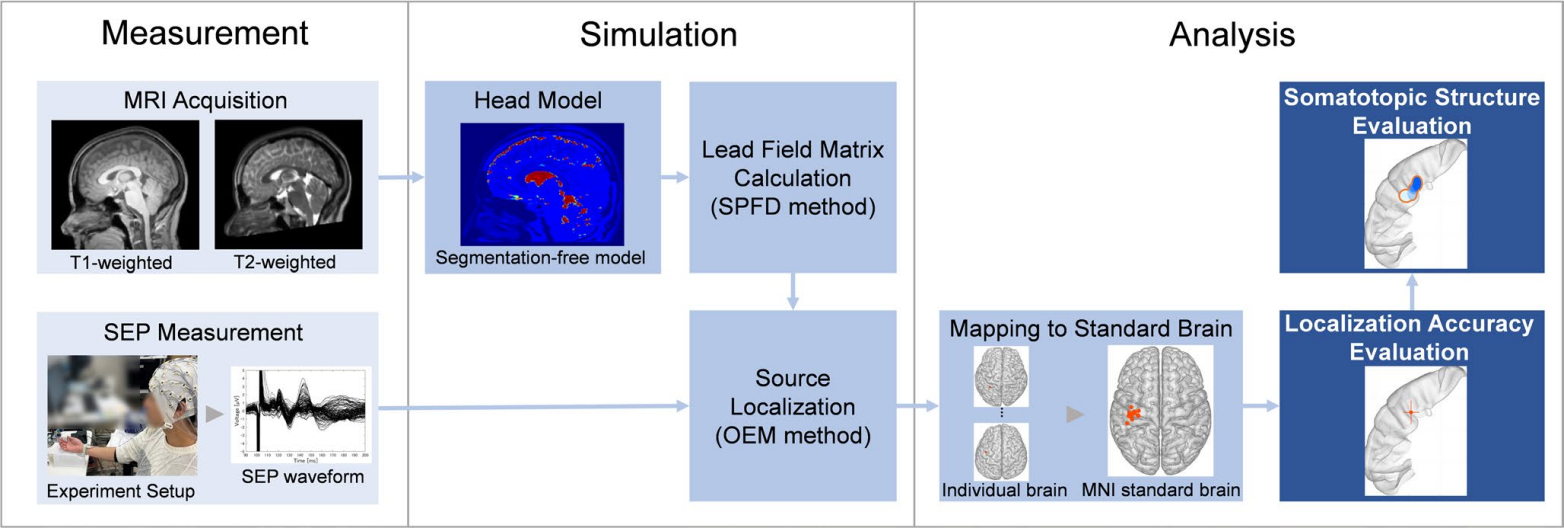
Overall methodological framework of the proposed source localization and analysis pipeline. The workflow consists of four main stages: (i) SEP measurement using a 65-channel EEG during peripheral nerve and finger stimulation; (ii) construction of personalized head models (segmented and segmentation-free models) from T1 and T2-weighted MRI; (iii) calculation of the lead-field matrix (LFM) using the scalar-potential finite-difference (SPFD) method; and (iv) source localization of the P20/N20 components using orthogonal matching pursuit (OMP). The results are then compared with the conventional finite element method (FEM) and standardized low-resolution electromagnetic tomography (sLORETA) pipeline to evaluate somatotopic consistency in the primary somatosensory cortex

The methodological framework employed in this study follows our previous report (Hirata et al. 2024); therefore, only a concise summary is provided. The present work extends the earlier framework in two important ways: (i) it is applied to multiple peripheral nerve and finger-stimulation targets to assess the effective spatial resolution of EEG-based source localization, and (ii) it incorporates a somatotopic analysis to determine whether finger-wise cortical representations can be resolved using EEG combined with personalized segmentation-free head models. An overview of the workflow, including these extensions, is presented in Fig. 1.

### Subjects

Sixteen healthy volunteers (mean age 22.5 ± 0.5 years) participated after providing written informed consent. The study protocol was approved by the Ethics Committees of the Nagoya Institute of Technology (Approval No. 2024-21) and the National Institute for Physiological Sciences (Approval No. EC01-098), and conducted in accordance with institutional guidelines and national regulations.

### Somatosensory Evoked Potentials Measurements

EEG signals were recorded using a 65-channel electrode cap (QuickAmp 72; Brain Products) following the International 10/10 system (Jurcak et al. 2007). The electrophysiological signals were recorded using an average reference, calculated as the mean potential across all electrodes, and data were re-referenced offline to a common average reference.

The EEG data were sampled at 2 kHz. A band-pass filter of 3–299 Hz was applied, together with notch filters at 60 Hz and its harmonics (120, 180, and 240 Hz) to suppress power-line interference. To minimize contamination from stimulation artifacts, data segments within the first 3 ms following stimulation onset were excluded from further analysis.

Each dataset was segmented into 500-ms epochs including a 100-ms pre-stimulus baseline period. Baseline correction was applied using the pre-stimulus interval. Channels exhibiting persistent artifacts were interpolated using spherical spline interpolation (if applicable). The cleaned epochs were then averaged across trials after aligning them to the stimulation onset to obtain the SEP waveforms. Source localization was performed using the time window centered at 20 ms post-stimulus (± 3 ms), based on the typical P20/N20 latency and allowing for inter-subject variability. All preprocessing parameters were kept identical across stimulation conditions to ensure comparability.

Electrical stimulation was delivered using a constant-current stimulator (SEN-8203, Nihon Koden). Monophasic-square pulses of 0.2 ms duration were applied at an inter-stimulus interval of 500 ms (2 Hz). Surface Ag/AgCl electrodes (NM-422B, Nihon Koden) were positioned over the median nerve at the wrist and the ulnar nerve at the wrist. Electrical stimulation was applied with the cathode placed 2 cm proximal to the anode. For finger stimulation, ring electrodes (NM-450 S Nihon Koden) were used, with the cathode placed proximal to the proximal interphalangeal joint and the anode distal to it.

For median and ulnar nerve stimulation, the current intensity was gradually increased until a slight visible motor twitch was observed. For individual finger stimulation, the sensory threshold was determined using an ascending–descending procedure. The current intensity was initially set at 3 mA and increased in 0.1 mA increments until the participant first perceived the stimulus. Subsequently, the current was decreased in 0.1 mA steps starting from a clearly perceived intensity, and the value immediately before the sensation disappeared was identified. The sensory threshold was defined as the average of these measurements. Stimulation was delivered at approximately 2–3 times this threshold. Across subjects, the mean stimulation intensity was 10.5 ± 2.5 mA for nerve stimulation and 13.1 ± 2.8 mA for finger stimulation.

### Source Localization using Personalized Head Models

The methodological framework used in this study follows our earlier detailed report and its simplified variant, with a different focus. Only the elements relevant to the present multitarget SEP analysis are summarized. Personalized head models derived from MRI scans were used to perform source localization and electric field computation following previously established procedures. In the present study, the term “personalized” refers to subject-specific head models constructed from each participant’s individual MRI data, including individualized anatomical geometry and voxel-wise conductivity estimation, rather than experimentally validated subject-specific conductivity ground truth. Anatomically detailed numerical head models were generated from T1- and T2-weighted MRI using advanced image-processing techniques, and these models formed the basis for computing intracranial electric fields and constructing the lead-field matrix (LFM), both of which are essential for reliable source localization in EEG.

The inverse problem was solved using the orthogonal matching pursuit (OMP) algorithm, which is a sparse modeling framework that iteratively selects the most plausible sources from a precomputed dictionary. Compared with conventional distributed inverse methods, OMP offers substantially greater computational efficiency while preserving high localization accuracy, making it suitable for repeated analyses across multiple stimulation targets. These properties underscore its utility as a robust alternative for EEG source estimation.

#### Head Model Construction

For each participant, high-resolution T1- and T2-weighted MR images (0.8-mm isotropic; Siemens Magnetom Verio) were acquired (T1w: 3D MPRAGE; T2w: 3D SPACE). All images were resampled to a 0.5-mm isotropic resolution using three-dimensional interpolation prior to head model generation. Two types of numerical head models were prepared.

1. **Segmented models**: canonical multi-tissue volume conductors in which each tissue is assigned a homogeneous dielectric property. The conductivities of 13 tissues (excluding the skin) were defined using the 4-Cole–Cole dispersive model (Gabriel et al. 1996), and the skin conductivity was set to 0.1 S/m to avoid overestimating the contribution of the stratum corneum.
2. **Segmentation-free models**: voxel-wise conductivity maps estimated directly from co-registered T1/T2 images using CondNet-TART (Rashed et al. 2020a; Kubota et al. 2025), a CNN-based estimator trained on paired MR volumes and conventional segmented conductors. This approach produces smoothly varying conductivity distributions across tissue boundaries, suppressing artificial field jumps at sharp interfaces (e.g., gray matter–CSF) that are inherent to piecewise constant segmentation. The native output resolution of CondNet-TART (1.0 mm isotropic) was resampled to 0.5 mm using trilinear interpolation to match the forward solver grid. These segmentation-free models preserve the CSF geometry at an anatomically detailed and more faithfully reflect the spatial variations associated with the tissue water content and microstructural features.

All MRI preprocessing steps, including resampling and co-registration of T1- and T2-weighted images, were performed using in-house scripts implemented in MATLAB (MathWorks, Natick, MA) and Python-based image-processing libraries. The pretrained CondNet-TART model was used as described in Kubota et al. (2025). The gray-matter source space was defined voxel-wise within the gray-matter mask, and three orthogonal dipole components were assigned at each voxel location. Prior systematic analyses have indicated that localization performance is relatively stable for model resolutions of approximately 1 mm or finer (Niitsu et al. 2026).

MRI scans were acquired at the National Institute for Physiological Sciences (Okazaki, Japan). Subsequent steps, including mesh-free voxel modeling, conductivity assignment, and quality checks for image registration and artifacts, followed our previously reported pipeline.

#### Finite Difference Method

The forward problem was solved using the scalar-potential finite-difference (SPFD) method (Dawson and Stuchly 1996). Under the quasi-static assumption appropriate for SEP timescales, the governing equation is the Poisson form

1 $$\:\begin{array}{c}\nabla\:\cdot \:\left({\upsigma\:}\nabla\:\phi\:\right)=-\nabla\:\cdot\:j,\end{array}$$

where *σ* is the conductivity, *ϕ* is the electric potential, and ***j*** is the applied current density. The potentials at the voxel nodes were treated as unknowns and assembled into a sparse linear system using finite-difference discretization on a 0.5-mm grid.

We employed successive over-relaxation (Hadjidimos 2000) using the geometric multigrid method (Laakso and Hirata 2012) to accelerate convergence. Six multi-grid levels were used, and the iterations were continued until the relative residual fell below 10^− 6^. A nominal driving frequency of 10 Hz (within the alpha band) was used only to parameterize the quasistatic solver; the field solutions were effectively frequency independent in this regime. Boundary conditions were imposed by designating a reference (ground) electrode and enforcing net current constraints associated with the stimulation/injection configurations described below.

Using *φ* the obtained from (1), the current density is derived as follows:

2 $$\:\begin{array}{c}j=\sigma\:E=-\sigma\:\nabla\:\phi\:,\end{array}$$

where ***E*** denotes the electric field induced in the head tissue.

#### Calculation of the LFM

The LFM links the cortical current sources to the measured scalp potentials. Let *M* denote the number of electrodes and *N* the number of gray-matter voxels; stacking the three Cartesian components per voxel yields a *M*×3*N* matrix *L* that maps the source vector ***j*** ∈ ℝ³ᴺ to the electrode potential vector *ϕ* ∈ ℝᴹ, expressed as *ϕ* = *L* ***j***.

The direct (brute-force) construction of *L* by exciting every candidate dipole is computationally prohibitive at a 0.5-mm resolution (millions of voxels). Therefore, we formed *L* using the reciprocity principle (Hallez et al. 2007; Weinstein et al. 2000) for each measurement electrode *m* (with fixed reference). A unit current *I* was injected between *m* and the reference, the SPFD was run once, and the induced electric field ***E***_m_(**r**) within the grey matter was recorded. The *x*, *y*, and *z* components of ***E***_m_ populate the corresponding L/I row blocks. Hence, only *M* forward solutions are required, while still accounting for the full 3-D source orientations.

No additional spatial smoothing or depth-weighting was applied to the forward model, ensuring that the lead-field matrix directly reflected the underlying conductivity distributions.

#### Inverse Problem

Source localization from the averaged SEP potentials focused on the P20/N20 time window. We assumed that the generators were focal (Fuchs et al. 1998; Aydin et al. 2014) and sparse, and therefore adopted the OMP (Pati et al. 1993).

Source orientations were not constrained a priori; instead, three orthogonal dipole components were considered at each voxel. The sparsity level (K = 5) was selected based on physiological considerations of focal SEP generators and preliminary stability analyses. All inverse computations were implemented using custom MATLAB scripts.

Starting from the measured scalp vectors *ϕ* and *L*, OMP iteratively.

1. selects the column (dipole basis) that is most closely correlated with the current residual.
2. updates the source coefficients via least squares on the active set.
3. refreshes the residual; and.
4. repeats until a fixed budget of components (K = 5) is reached, or the residual reduction saturates.

The output was a set of up to five source locations with associated weights. Because the OMP coefficients approximate dipole moments rather than a full vector field on the cortex, we reconstructed the cortical current density by rerunning the SPFD with elementary dipoles at the OMP-selected locations and forming their weighted superposition. The composite distribution within the grey matter was used for subsequent accuracy and between-method comparisons. This two-stage (sparse selection → field reconstruction) strategy retains computational efficiency while providing physically interpretable current maps.

### Analysis with sLORETA using Brainstorm

To provide a benchmark for our proposed sparse estimation method, we employed the open-source Brainstorm toolbox (https://neuroimage.usc.edu/brainstorm/Introduction) developed in MATLAB, which is widely used for EEG data analyses (Tadel et al. 2011). Brainstorm implements several standard head-modeling approaches, including the following:

1. a three-layer spherical conductor model,
2. boundary element models (BEM) using OpenMEEG, and.
3. finite element models (FEM) using DUNEuro (Medani et al. 2023).

These complementary models enable comparisons across levels of anatomical fidelity and numerical complexity.

For the inverse problem, we applied standardized low-resolution electromagnetic tomography (sLORETA), a widely used distributed-source localization algorithm (Pascual-Marqui 2002). In line with common practice, we defined a grid of approximately 60,000 candidate dipoles distributed across the cortical mantle. The current density distribution was first estimated using the minimum-norm (MN) approach (Hämäläinen and Ilmoniemi 1994; Grech et al. 2008; Asadzadeh et al. 2020) and then standardized using sLORETA to reduce depth bias and improve comparability across subjects.

### Evaluation of Somatotopic Organization

To evaluate whether the estimated source locations exhibited somatotopic organization within the primary somatosensory cortex, mean source coordinates were computed for each stimulation target (median nerve, ulnar nerve, and each finger). For finger stimulations, the mean coordinates and their across-subject variability were visualized as ellipsoids centered at the mean positions, with diameters representing one standard deviation.

To assess spatial ordering, the mean coordinates were plotted as a function of finger identity (from the thumb to the little finger) on the coronal and axial planes using a consistent color scheme across the stimulation targets. Linear trends along the lateral–medial and superior–inferior axes were evaluated to determine whether the estimated sources followed the expected somatotopic progression in the left hemisphere.

### Statistical Analysis

Linear regressions and Pearson correlation coefficients were computed to assess the relationship between finger identity and estimated source coordinates. Statistical significance was evaluated using two-tailed tests with a threshold of *p* < 0.05. No correction for multiple comparisons was applied because the analyses were restricted to two predefined anatomical axes (lateral–medial and superior–inferior).

## Results

### Estimation Accuracy

**Fig. 2:**
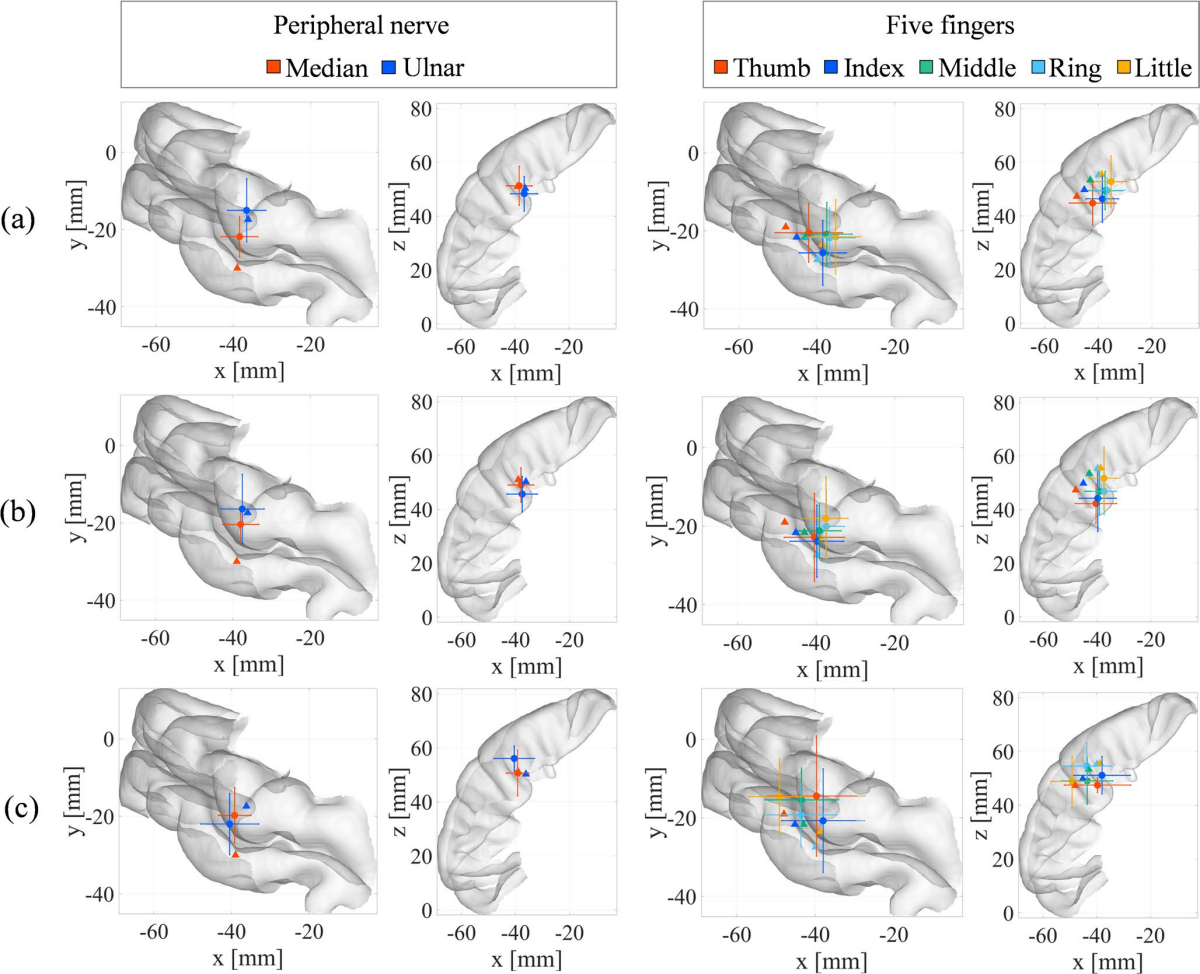
Representative source localization results for peripheral nerve and finger stimulation. The figure is organized into three rows and two columns. Rows represent the source estimation methods: (**a**) the segmentation-free model with OMP, (**b**) the segmented model with OMP, and (**c**) the FEM-based model with sLORETA. Columns represent the stimulation targets: peripheral nerve stimulation (median and ulnar nerves; left column) and five-finger stimulation (thumb, index, middle, ring, and little fingers; right column). Each point indicates the mean source coordinate, and the bars denote the standard deviation across the subjects. The corresponding Euclidean distances are summarized in Table 1

Table 1 summarizes the Euclidean distances between the estimated source coordinates obtained by each method and the reference MNI coordinates for the median (Bingel et al. 2004), ulnar (Houzé et al. 2011), and finger stimulations (Holmes and Tamè 2019). Figure 2 shows the representative localization results for each stimulation target, complementing the quantitative results in Table 1. The corresponding averaged scalp-level SEP waveforms and topographical distributions for each stimulation condition are provided in Supplementary Fig. S1 and Fig. S2, respectively, confirming that the observed source differences are supported by measurable differences in the recorded EEG signals. Across all stimulation conditions, the segmentation-free model consistently produced smaller source-location differences than the segmented model, with reductions in localization differences of up to 2.9 mm.

Compared with the FEM-based forward model combined with sLORETA, the segmentation-free model with OMP also showed reduced localization differences for all stimulation targets, except for the ring finger. The segmented model demonstrated intermediate performance, falling between the segmentation-free and FEM+sLORETA approaches.

For peripheral nerve stimulation (median and ulnar nerves), the localization differences were approximately 1–5 mm smaller than those for finger stimulation, although the overall effect of the stimulation type on localization accuracy was relatively modest.

**Table 1:** Localization accuracy of the three source-estimation methods for each stimulation target. Each value represents the mean ± SD of the Euclidean distances between the estimated and reference MNI coordinates

Euclidean distance (mm)			
Nerve	(a) Segmentation-free Model + OMP	(b) Segmented Model + OMP	(c) FEM mesh Model + sLORETA
Median	12.6 ± 3.0	13.0 ± 2.8	14.5 ± 5.8
Ulnar	10.4 ± 5.9	11.7 ± 6.4	13.9 ± 4.4
Thumb	14.9 ± 4.6	17.0 ± 6.6	19.8 ± 9.4
Index	15.1 ± 5.1	18.0 ± 5.0	19.0 ± 4.7
Middle	13.2 ± 5.5	14.0 ± 4.0	16.1 ± 5.2
Ring	13.8 ± 3.0	16.7 ± 5.9	16.3 ± 6.0
Little	14.4 ± 6.3	16.3 ± 7.7	20.2 ± 8.2

### Somatotopic Organization of Estimated Sources

**Fig. 3:**
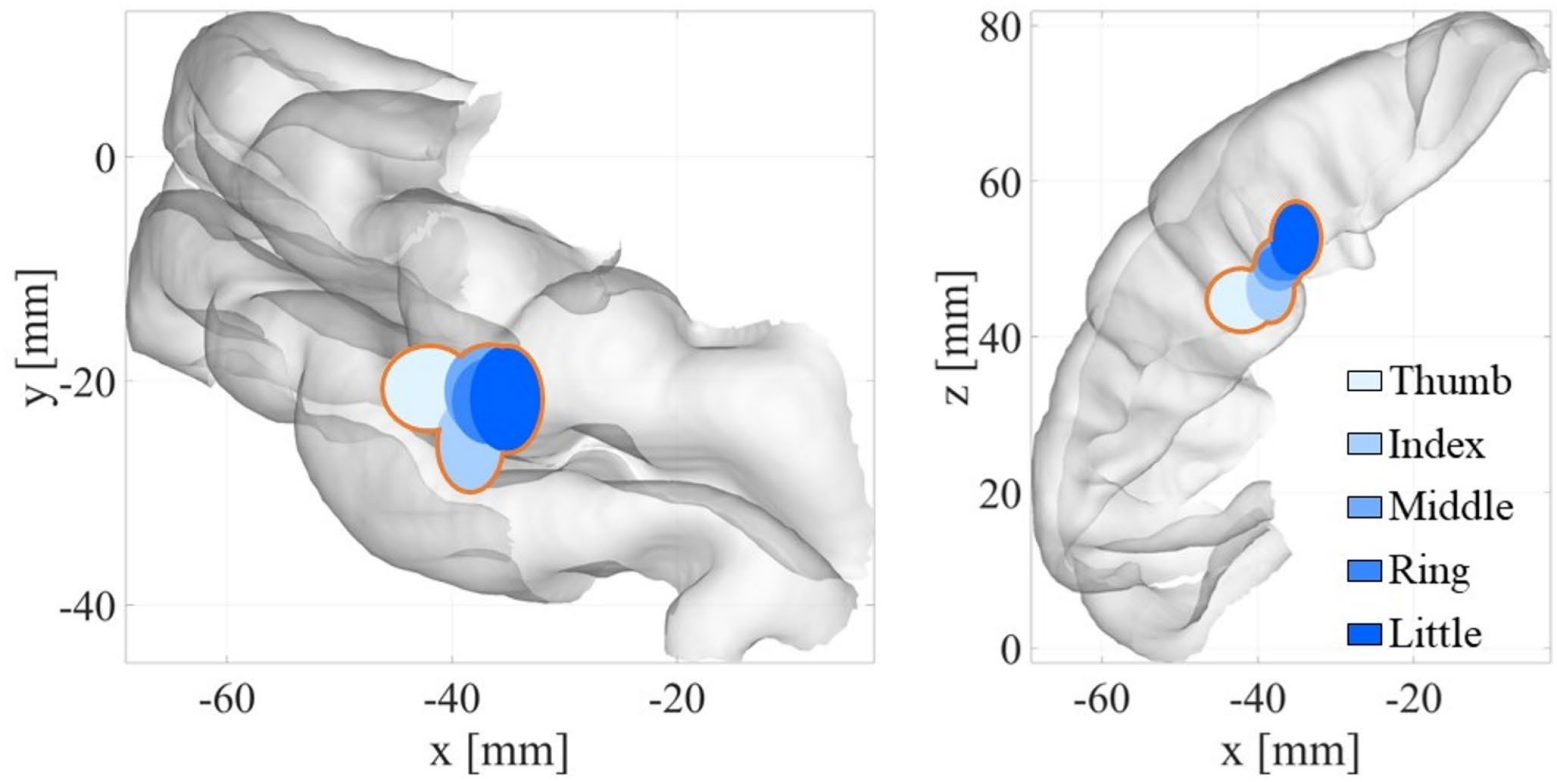
Somatotopic organization of estimated sources using the segmentation-free model with the OMP method for five-finger stimulation. Each ellipsoid is centered at the mean source coordinate, and its diameter represents the standard deviation across subjects

**Fig. 4:**
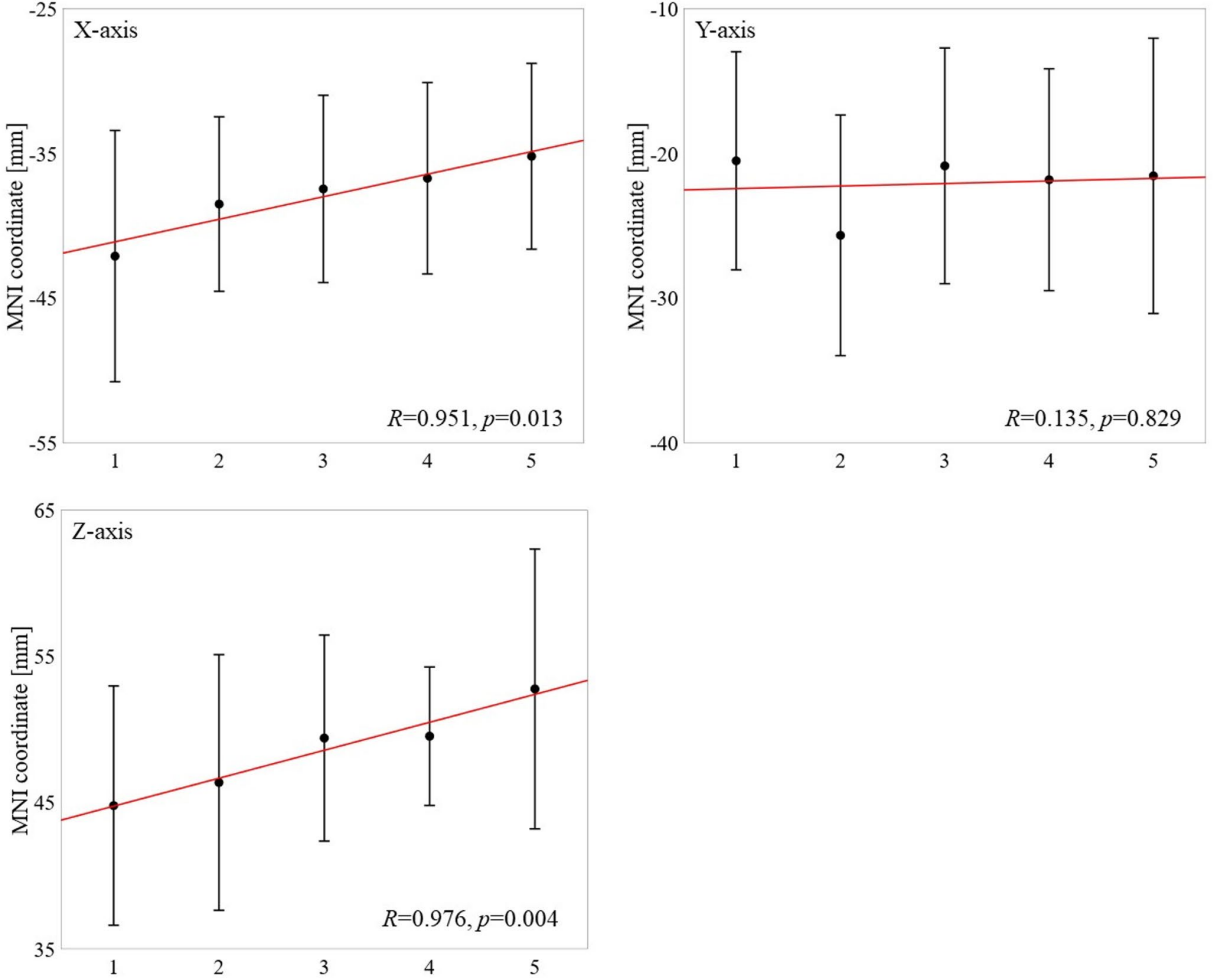
Correlation between the finger-stimulation order and mean source coordinates. The numbers on the horizontal axis (1–5) correspond to the thumb, index, middle, ring, and little fingers, respectively. Each point denotes the mean source coordinate across the subjects, with error bars indicating the standard deviation. The red lines represent fitted linear regressions

Figure 3 shows the estimated source distributions for the five finger stimulations (thumb, index, middle, ring, and little fingers). A clear somatotopic progression was evident in the coronal plane, with the source locations shifting from the lateral (thumb) to the medial (little finger) side. Figure 4 further illustrates the systematic changes in the mean source coordinates as a function of the finger identity. Significant linear relationships were observed along both the x-axis (*R* = 0.951, *p* < 0.05) and z-axis (*R* = 0.976, *p* < 0.05), demonstrating orderly spatial separation across the fingers.

Across all finger stimulations, the estimated sources were consistently located deeper than those reported in previous fMRI studies. Although the magnitude of this depth difference varied among the fingers, the overall trend was consistent across the subjects.

## Discussion

The central premise of the present study is that the fidelity of the forward electromagnetic model fundamentally constrains the achievable stability and consistency of EEG source localization. This study demonstrated that incorporating voxel-wise heterogeneous conductivities into personalized head models improved the spatial consistency of EEG-based source localization across stimulation targets. This improvement stems from the segmentation-free approach, which provides continuous conductivity transitions that minimize artificial boundary effects and enhance the conditioning of the forward problem, yielding smoother conductivity transitions and reducing artificial boundary discontinuities under the adopted modeling assumptions. When combined with a sparsity-promoting inverse solver, such as OMP, these biophysically informed models directly contribute to more spatially consistent source estimates.

Previous neuroimaging studies have established that the human primary somatosensory cortex exhibits an orderly somatotopic organization, with finger representations progressing from the thumb to the little finger in lateral–medial and superior–inferior gradients (Jamali and Ross 2013; Janko et al. 2022). Related studies have also demonstrated somatotopic organization of nociceptive processing (Bingel et al. 2004). However, most prior mappings relied on MEG, fMRI, or simplified head models, and few investigations have tested whether EEG combined with anatomically detailed forward models can resolve somatotopy at the level of individual fingers.

Our results show that a segmentation-free, voxel-wise conductivity model combined with OMP successfully reconstructs orderly finger representations from EEG-derived SEPs, demonstrating that detailed somatotopic mapping is achievable without MEG or fMRI during measurements. This finding represents a meaningful methodological advance, given the accessibility, portability, and clinical practicality of EEG.

It is important to distinguish between absolute localization accuracy and relative somatotopic consistency. Demonstrating a consistent lateral–medial and superior–inferior progression does not necessarily imply improved absolute localization accuracy, as systematic spatial offsets may persist across methods. Accordingly, the primary finding of this study is improved spatial consistency across stimulation targets under refined forward-model assumptions rather than a definitive enhancement of intrinsic EEG spatial resolution.

The estimated finger sources were consistently deeper than the reference coordinates reported in previous fMRI studies. This deviation likely reflects physiological differences in the activated cortical layers: fMRI-based coordinates typically correspond to Brodmann area 3b, which primarily receives cutaneous input, whereas the 2–3× sensory-threshold stimulation used in this study may have recruited deeper generators in area 3a, which is associated with proprioceptive and mixed sensory processing. These observations highlight the influence of stimulation parameters on SEP localization and suggest that adjusting the stimulus intensity may enable more targeted probing of distinct somatosensory subregions.

These depth-related findings are also broadly in line with recent ultra–high-field layer-fMRI studies, which reported that conventional BOLD signals predominantly reflect activity in superficial cortical layers, whereas sensory inputs in area 3b are most prominent in middle layers (layer 4) (Yu et al. 2019). Although the present EEG analysis does not resolve laminar profiles directly, segmentation-free head modeling in subject-specific anatomy could, in principle, be combined with layer-fMRI acquired in native space to improve depth-specific validation of EEG source estimates. Such multimodal integration may help clarify how laminar activation patterns translate into depth-dependent sensitivity in noninvasive electrophysiological source imaging.

It is important to clarify the scope of the present contribution. Segmentation-free conductivity modeling and sparse inverse estimation have been previously described in methodological studies. The present work extends those approaches by systematically evaluating their performance across multiple somatosensory stimulation targets within the same cohort. In particular, we demonstrate that forward-model refinement improves spatial consistency across median nerve, ulnar nerve, and individual finger stimulations under identical inverse assumptions.

Moreover, the observed orderly somatotopic progression should be interpreted as evidence of relative spatial consistency rather than definitive proof of absolute localization accuracy. Because reference coordinates were derived from prior neuroimaging studies rather than subject-specific ground truth, systematic spatial offsets cannot be excluded. Nonetheless, the consistent reduction in localization differences between segmentation-free and segmented models suggests that improving forward-model realism reduces structured lead-field bias, thereby enhancing the stability of inverse estimates.

Inter-subject anatomical variability may also contribute to the residual localization differences observed in this study. Individual variations in cortical folding patterns, skull thickness, and conductivity distributions can introduce subject-specific spatial offsets relative to literature-based MNI coordinates. Therefore, part of the deviation from reference coordinates may reflect genuine anatomical variability rather than modeling inaccuracy alone.

Furthermore, the reference coordinates used for comparison were derived from previously published neuroimaging studies rather than subject-specific ground truth. The present findings therefore emphasize relative improvements across modeling frameworks rather than intrinsic spatial resolution limits of EEG.

The choice of inverse solver should also be interpreted in the context of the present experimental design. Early SEP components such as P20/N20 are predominantly generated by focal cortical sources, which makes sparse source modeling physiologically plausible. In this study, OMP was employed under the assumption of limited and focal generators.

Recent inverse algorithms such as FAST-IRES (Sohrabpour et al. 2020) and STSI (Jiang et al. 2025) have demonstrated strong performance in epilepsy source localization validated against intracranial recordings. These approaches are particularly suited for complex, potentially distributed spike generators recorded with high-density EEG. In contrast, the present study focuses on stimulus-locked SEP responses acquired with moderate-density EEG, where sparse modeling assumptions remain appropriate.

The comparison between segmentation-free and segmented head models was performed using the same inverse solver, thereby isolating the effect of forward-model refinement. Improvements in forward modeling reduce systematic bias in the lead-field matrix, which cannot be fully compensated by inverse regularization alone. Therefore, the observed improvement in spatial consistency primarily reflects enhanced forward-model realism rather than superiority of a specific inverse algorithm.

A previous study (Lee et al. 2009) comparing different conductivity modeling strategies has reported relatively modest effects on absolute EEG source localization accuracy when evaluated against fMRI-based references. Therefore, the present findings should be interpreted primarily as improvements in relative spatial consistency across stimulation targets under identical inverse assumptions, rather than definitive evidence of substantially enhanced absolute localization accuracy.

This study has several limitations. First, the number of participants (*n* = 16) was relatively small, which may limit generalizability; larger cohorts will be necessary to confirm the reproducibility of the observed somatotopic patterns in future studies. Second, it is important to distinguish between absolute localization accuracy and relative somatotopic consistency. In the present study, localization differences were quantified relative to previously reported MNI coordinates, whereas somatotopic organization was evaluated based on the spatial ordering of finger representations. Demonstrating a consistent lateral–medial and superior–inferior progression does not necessarily imply improved absolute localization accuracy, as systematic spatial offsets may persist across methods. Third, although voxel-wise modeling improved localization accuracy, a slight depth bias remained, particularly with stronger stimulation. Future studies should explore stimulation paradigms that better balance superficial and deep generators. Fourth, extending this framework to multisource conditions, incorporating anisotropic conductivity models, and pursuing real-time implementations would broaden its applicability to both basic neuroscience and clinical practice.

In addition, the voxel-wise conductivity distributions used in the present study were estimated using an MRI-based computational framework and were not validated against experimental in vivo conductivity ground truth. Therefore, the present findings should be interpreted as relative improvements in spatial consistency and forward-model behavior under the proposed modeling assumptions, rather than definitive validation of absolute conductivity distributions or localization accuracy.

Accordingly, the present findings should be interpreted primarily as evidence of improved relative spatial consistency across stimulation targets under controlled modeling assumptions, rather than definitive validation of intrinsic EEG spatial resolution or absolute source localization accuracy.

In summary, combining a segmentation-free voxel-wise conductivity model with sparse inverse estimation provides a promising pathway for improving spatial consistency in noninvasive functional brain mapping using EEG. The demonstrated ability to reconstruct orderly finger representations underscores the potential of this approach for studying fine-scale somatosensory organization and supporting future neurophysiological and clinical applications of the method. Such improved spatial consistency may further benefit presurgical sensory mapping, longitudinal neurorehabilitation monitoring, and refined decoding strategies for brain–machine interfaces.

## Conclusion

This study demonstrated that the combination of a personalized segmentation-free head model and an OMP-based inverse solver enables spatially consistent EEG source localization of somatosensory evoked potentials. The segmentation-free approach reduced localization differences across multiple stimulation targets, including the median nerve, ulnar nerve, and individual fingers, by providing voxel-wise heterogeneous conductivity distributions that minimized artificial discontinuities at tissue boundaries. To the best of our knowledge, this is one of the first studies to show that a segmentation-free forward model can reproduce the somatotopic organization of the primary somatosensory cortex based on EEG measurements. The estimated sources for finger stimulation followed a continuous lateral-to-medial and superior-to-inferior progression, consistent with known cortical representations, demonstrating the ability of this approach to noninvasively resolve fine-scale somatotopic organization. These findings highlight the potential of segmentation-free modeling for spatially consistent electrophysiological source imaging. Future studies should extend this framework to larger cohorts and optimize stimulation protocols to further enhance its applicability in clinical and neurophysiological settings.

## Supplementary Information

Below is the link to the electronic supplementary material.

Supplementary Material 1

## Data Availability

The datasets generated and/or analyzed during the current study are not publicly available due to ethical and privacy restrictions related to human participant data, but are available from the corresponding author upon reasonable request.

## References

[CR1] An N, Cao F, Li W, Wang W, Xu W, Wang C et al (2022) Spatial accuracy evaluation of magnetic source imaging methods on OPM-based MEG. iScience 25(10):105177. 10.1016/j.isci.2022.105177 36238897 10.1016/j.isci.2022.105177 PMC9550608

[CR2] Asadzadeh S, Rezaii TY, Beheshti S, Delpak A, Meshgini S (2020) A systematic review of EEG source localization techniques and their applications on diagnosis of brain abnormalities. J Neurosci Methods 339:108740. 10.1016/j.jneumeth.2020.108740 32353472 10.1016/j.jneumeth.2020.108740

[CR3] Aydin Ü, Vorwerk J, Küpper P, Heers M, Kugel H, Galka A et al (2014) Combining EEG and MEG for the reconstruction of epileptic activity using a calibrated realistic volume conductor model. PLoS ONE 9(3):e93154. 10.1371/journal.pone.0093154 24671208 10.1371/journal.pone.0093154 PMC3966892

[CR4] Bergamaschi R, Romani A, Versino M, Callieco R, Sartori I, Cosi V (1993) Somatosensory evoked potentials by median nerve stimulation recorded with cephalic and non-cephalic references. II). Reliability study. Boll Soc Ital Biol Sper 69(9):533–539 8155311

[CR5] Bingel U, Lorenz J, Glauche V, Knab R, Gläscher J, Weiller C et al (2004) Somatotopic organization of human somatosensory cortices for pain: a single trial fMRI study. NeuroImage 23(1):224–232. 10.1016/j.neuroimage.2004.05.021 15325369 10.1016/j.neuroimage.2004.05.021

[CR6] Connemann B, Koehler J, Presser S, Hopf H (1999) Latency and amplitude variability in serial median nerve SEP recordings. Clin Neurophysiol 110(9):1664–1668. 10.1016/s1388-2457(99)00096-6 10479037 10.1016/s1388-2457(99)00096-6

[CR7] Dawson TW, Stuchly MA (1996) Analytic validation of a three-dimensional scalar-potential finite-difference code for low-frequency magnetic induction. Appl Comput Electromagnet J 11:72–81

[CR8] Depuydt E, Criel Y, De Letter M, Van Mierlo P (2024) Investigating the effect of template head models on Event-Related Potential source localization: a simulation and real-data study. Front NeuroSci 18:1443752 39440187 10.3389/fnins.2024.1443752 PMC11493687

[CR9] Ebersole J (1994) Non-invasive localization of the epileptogenic focus by EEG dipole modeling. Acta Neurol Scand 89(S152):20–28. 10.1111/j.1600-0404.1994.tb05179.x 10.1111/j.1600-0404.1994.tb05179.x 8209647

[CR10] Fuchs M, Wagner M, Wischmann H-A, Köhler T, Theißen A, Drenckhahn R et al (1998) Improving source reconstructions by combining bioelectric and biomagnetic data. Electroencephalogr Clin Neurophysiol 107(2):93–111. 10.1016/s0013-4694(98)00046-7 9751281 10.1016/s0013-4694(98)00046-7

[CR11] Gabriel S, Lau RW, Gabriel C (1996) The dielectric properties of biological tissues: III. Parametric models for the dielectric spectrum of tissues. Phys Med Biol 41(11):2271–2293. 10.1093/brain/114.6.2465 8938026 10.1088/0031-9155/41/11/003

[CR12] Grech R, Cassar T, Muscat J, Camilleri KP, Fabri SG, Zervakis M et al (2008) Review on solving the inverse problem in EEG source analysis. J Neuroeng Rehabilitation 5(1):1–33. 10.1186/1743-0003-5-25 10.1186/1743-0003-5-25 PMC2605581 18990257

[CR13] Hadjidimos A (2000) Successive overrelaxation (SOR) and related methods. J Comput Appl Math 123(1–2):177–199. 10.1016/S0377-0427(00)00403-9

[CR14] Hallez H, Vanrumste B, Grech R, Muscat J, De Clercq W, Vergult A et al (2007) Review on solving the forward problem in EEG source analysis. J Neuroeng Rehabil 4(1):1–29. 10.1186/1743-0003-4-46 18053144 10.1186/1743-0003-4-46 PMC2234413

[CR15] Hämäläinen MS, Ilmoniemi RJ (1994) Interpreting magnetic fields of the brain: minimum norm estimates. Med Biol Eng Comput 32:35–42. 10.1007/bf02512476 8182960 10.1007/BF02512476

[CR16] Hari R, Forss N (1999) Magnetoencephalography in the study of human somatosensory cortical processing. Philosophical Trans Royal Soc Lond Ser B: Biol Sci 354(1387):1145–1154. 10.1098/rstb.1999.04701145-1154 10.1098/rstb.1999.0470 PMC1692629 10466142

[CR17] Hirata A, Niitsu M, Phang CR, Kodera S, Kida T, Rashed EA et al (2024) High-resolution EEG source localization in personalized segmentation-free head model with multi-dipole fitting. Phys Med Biol 69(5):055013. 10.1088/1361-6560/ad25c3 10.1088/1361-6560/ad25c3 38306964

[CR18] Holmes NP, Tamè L (2019) Locating primary somatosensory cortex in human brain stimulation studies: systematic review and meta-analytic evidence. J Neurophysiol 121(1):152–162. 10.1152/jn.00614.2018 30517062 10.1152/jn.00614.2018

[CR19] Houzé B, Perchet C, Magnin M, Garcia-Larrea L (2011) Cortical representation of the human hand assessed by two levels of high‐resolution EEG recordings. Hum Brain Mapp 32(11):1894–1904. 10.1002/hbm.21155 21246666 10.1002/hbm.21155 PMC6870146

[CR20] Jamali S, Ross B (2013) Somatotopic finger mapping using MEG: toward an optimal stimulation paradigm. Clin Neurophysiol 124(8):1659–1670. 10.1016/j.clinph.2013.01.027 23518470 10.1016/j.clinph.2013.01.027

[CR21] Janko D, Thoenes K, Park D, Willoughby W, Horton M, Bolding M (2022) Somatotopic mapping of the fingers in the somatosensory cortex using functional magnetic resonance imaging: A review of literature. Front Neuroanat 16:866848. 10.3389/fnana.2022.866848 35847829 10.3389/fnana.2022.866848 PMC9277538

[CR22] Ji Y, Meng B, Yuan C, Yang H, Zou J (2013) Monitoring somatosensory evoked potentials in spinal cord ischemia-reperfusion injury. Neural Regeneration Res 8(33):3087–3094. 10.3969/j.issn.1673-5374.2013.33.002 10.3969/j.issn.1673-5374.2013.33.002 PMC4158706 25206629

[CR23] Jiang X, Cai Z, Gonsisko C, Worrell GA, He B (2025) Mapping epileptogenic brain using a unified spatial–temporal–spectral source imaging framework. Proc Nat Acad Sci 122(50):e2510015122. 10.1073/pnas.2510015122 41359838 10.1073/pnas.2510015122 PMC12718345

[CR24] Jurcak V, Tsuzuki D, Dan I (2007) 10/20, 10/10, and 10/5 systems revisited: their validity as relative head-surface-based positioning systems. NeuroImage 34(4):1600–1611. 10.1016/j.neuroimage.2006.09.024 17207640 10.1016/j.neuroimage.2006.09.024

[CR25] Kubota Y, Kodera S, Hirata A (2025) A Novel Transfer Learning Framework for Non-Uniform Conductivity Estimation with Limited Data in Personalized Brain Stimulation. Phys Med Biol 70:105002. 10.1088/1361-6560/add105 10.1088/1361-6560/add105 40280154

[CR26] Laakso I, Hirata A (2012) Fast multigrid-based computation of the induced electric field for transcranial magnetic stimulation. Phys Med Biol 57(23):7753–7765. 10.1088/0031-9155/57/23/7753 23128377 10.1088/0031-9155/57/23/7753

[CR27] Lebedev MA, Nicolelis MA (2006) Brain–machine interfaces: past, present and future. Trends Neurosci 29(9):536–546. 10.1016/j.tins.2006.07.004 16859758 10.1016/j.tins.2006.07.004

[CR28] Lee WH, Liu Z, Mueller BA, Lim K, He B (2009) Influence of white matter anisotropic conductivity on EEG source localization: Comparison to fMRI in human primary visual cortex. Clin Neurophysiol 120(12):2071–2081 19833554 10.1016/j.clinph.2009.09.007 PMC2790013

[CR29] Medani T, Garcia-Prieto J, Tadel F, Antonakakis M, Erdbrügger T, Höltershinken M et al (2023) Brainstorm-DUNEuro: An integrated and user-friendly Finite Element Method for modeling electromagnetic brain activity. NeuroImage 267:119851. 10.1016/j.neuroimage.2022.119851 36599389 10.1016/j.neuroimage.2022.119851 PMC9904282

[CR30] Moridera T, Rashed EA, Mizutani S, Hirata A (2021) High-resolution EEG source localization in segmentation-free head models based on finite-difference method and matching pursuit algorithm. Front NeuroSci 15:695668. 10.3389/fnins.2021.695668 34262433 10.3389/fnins.2021.695668 PMC8273249

[CR31] Niitsu M, Kodera S, Kubota Y, Tada Y, Wasaka T, Hirata A (2026) Influence of personalized human head modeling and resolution on EEG source localization for rapid brain mapping. Phys Med Biol 71:035012. 10.1088/1361-6560/ae3cf7 10.1088/1361-6560/ae3cf7 41576536

[CR32] Pascual-Marqui RD (2002) Standardized low-resolution brain electromagnetic tomography (sLORETA): technical details. Methods Find Exp Clin Pharmacol 24(Suppl D):5–12 12575463

[CR33] Pati YC, Rezaiifar R, Krishnaprasad PS (1993) ‘Orthogonal matching pursuit: Recursive function approximation with applications to wavelet decomposition’ *Proc. 27th Asilomar Conf. Signals, Systems and Computers*. IEEE, pp. 40–44. Available at: 10.1109/ACSSC.1993.342465

[CR34] Perot PL Jr (1973) The clinical use of somatosensory evoked potentials in spinal cord injury. Neurosurgery 20:367–381. 10.1093/neurosurgery/20.cn_suppl_1.367 10.1093/neurosurgery/20.cn_suppl_1.367 4587102

[CR35] Rashed EA, Diao Y, Hirata A (2020a) Learning-based estimation of dielectric properties and tissue density in head models for personalized radio-frequency dosimetry. Phys Med Biol 65(6):065001. 10.1088/1361-6560/ab7308 32023556 10.1088/1361-6560/ab7308

[CR36] Rashed EA, Gomez-Tames J, Hirata A (2020b) Deep learning-based development of personalized human head model with non-uniform conductivity for brain stimulation. IEEE Trans Med Imaging 39(7):2351–2362. 10.1109/tmi.2020.2969682 31995479 10.1109/TMI.2020.2969682

[CR37] Rice JK, Rorden C, Little JS, Parra LC (2013) Subject position affects EEG magnitudes. NeuroImage 64:476–484. 10.1016/j.neuroimage.2012.09.041 23006805 10.1016/j.neuroimage.2012.09.041

[CR38] Sohrabpour A, Cai Z, Ye S, Brinkmann B, Worrell G, He B (2020) Noninvasive electromagnetic source imaging of spatiotemporally distributed epileptogenic brain sources. Nat Commun 11(1):1946. 10.1038/s41467-020-15781-0 32327635 10.1038/s41467-020-15781-0 PMC7181775

[CR39] Tadel F, Baillet S, Mosher JC, Pantazis D, Leahy RM (2011) Brainstorm: a user-friendly application for MEG/EEG analysis. *Computational Intelligence and Neuroscience*, 2011, 1–13. 10.1155/2011/879716 10.1155/2011/879716 PMC3090754 21584256

[CR40] Vorwerk J, Clerc M, Burger M, Wolters C (2012) Comparison of boundary element and finite element approaches to the EEG forward problem. Biomedical Engineering/Biomedizinische Technik 57(SI–1–Track–O):795–798. 10.1515/bmt-2012-4152 10.1515/bmt-2012-4152 23096316

[CR41] Weinstein D, Zhukov L, Johnson C (2000) Lead-field bases for electroencephalography source imaging. Ann Biomed Eng 28:1059–1065. 10.1114/1.1310220 11132189 10.1114/1.1310220

[CR42] Yu Y, Huber L, Yang J, Jangraw DC, Handwerker DA, Molfese PJ et al (2019) Layer-specific activation of sensory input and predictive feedback in the human primary somatosensory cortex. Sci Adv 5(5):eaav9053. 10.1126/sciadv.aav9053 31106273 10.1126/sciadv.aav9053 PMC6520017

[CR43] Yvert B, Bertrand O, Thevenet M, Echallier J, Pernier J (1997) A systematic evaluation of the spherical model accuracy in EEG dipole localization. Electroencephalogr Clin Neurophysiol 102(5):452–459. 10.1016/s0921-884x(97)96611-x 9191589 10.1016/s0921-884x(97)96611-x

