# Supplementary material for: Improved Somatotopic Consistency of EEG Source Localization Using a Personalized Segmentation-Free Head Model
Source: Brain Topogr. 2026 May 26;39(4):60. doi: 10.1007/s10548-026-01217-3 (PMC13212632; doi:10.1007/s10548-026-01217-3)
Supplement: Supplementary file 1 — Supplementary Material 1 [file 10548_2026_1217_MOESM1_ESM.docx]

**Supplementary Information**

This file contains supplementary figures for the article "Improved Somatotopic Consistency of EEG Source Localization Using a Personalized Segmentation-Free Head Model" by Tada Y et al.

**Fig. S1** Butterfly plots of averaged somatosensory evoked potentials for median nerve, ulnar nerve, and individual finger stimulation. The yellow shaded region indicates the P20/N20 time window (20 ± 3 ms post-stimulus) used for source localization.

**Fig. S2** Scalp topographies of SEP amplitudes within the P20/N20 time window for each stimulation condition.
